# A novel protective role of sacubitril/valsartan in cyclophosphamide induced lung injury in rats: impact of miRNA-150-3p on NF-κB/MAPK signaling trajectories

**DOI:** 10.1038/s41598-020-69810-5

**Published:** 2020-08-03

**Authors:** Ghada A. Abdel-Latif, Azza H. Abd Elwahab, Rehab A. Hasan, Noura F. ElMongy, Maggie M. Ramzy, Manal L. Louka, Mona F. Schaalan

**Affiliations:** 1 Pharmacology and Toxicology Department, Faculty of Pharmacy, Misr International University (MIU), KM 28, Cairo-Ismailia Road (Ahmed Orabi District), Cairo, Egypt; 2 Physiology Department, Faculty of Medicine for Girls, Al-Azhar University, Cairo, Egypt; 3 Histology Department, Faculty of Medicine for Girls, Al-Azhar University, Cairo, Egypt; 4 Physiology Department, Faculty of Medicine, Al-Azhar University, Damietta, Egypt; 5 Biochemistry Department, Faculty of Medicine, Minia University, Minya, Egypt; 6 Medical Biochemistry Department, Faculty of Medicine, Ain Shams University, Cairo, Egypt; 7 Pharmacy Practice and Clinical Pharmacy Department, Faculty of Pharmacy, Misr International University (MIU), Cairo, Egypt; 8 Translational and Clinical Research Unit, Faculty of Pharmacy, Misr International University (MIU), Cairo, Egypt

**Keywords:** Analytical biochemistry, Predictive markers, Translational research

## Abstract

Cyclophosphamide (CP) is a chemotherapeutic agent that induces oxidative stress causing multiple organ damage. Sacubitril/valsartan, is a combined formulation of neprilysin inhibitor (sacubitril) and angiotensin II receptor blocker (valsartan), that induces the protective effect of brain natriuretic peptide. The aim of the current study is to investigate the prophylactic impacts of sacubitril/valsartan versus valsartan against CP-induced lung toxicity in rats. Rats were assigned randomly into 6 groups; control; received corn oil (2 ml/kg/day; p.o. for 6 days), sacubitril/valsartan (30 mg/kg; p.o. for 6 days), valsartan (15 mg/kg; p.o. for 6 days), CP (200 mg/kg; i.p. on day 5), sacubitril/valsartan + CP (30 mg/kg; p.o. for 6 days, 200 mg/kg; i.p. single dose on day 5, respectively), valsartan + CP (15 mg/kg; p.o. for 6 days, 200 mg/kg; i.p. single dose on day 5, respectively). Both sacubitril/valsartan and valsartan produced a significant decrease in the inflammation and fibrosis markers in the BALF, in comparison with the CP group. Both sacubitril/valsartan and valsartan produced an apparent decrease in the relative genes expression of miR-150-3p and NF-κB, as well as a significant decrease in the relative expression of P38 and ERK1/2 MAPKs and an increase in the relative gene expression of Nrf-2, compared to CP group. Intriguingly, sacubitril/valsartan , showed subtle superiority in almost all investigated parameters, compared to valsartan. In conclusion, sacubitril/valsartan effectively abrogated the CP induced lung inflammation and fibrosis, providing a potential promising protection that could be linked to their ability to inhibit miR-150-3p via inhibition of NF-κB and MAPK signaling pathways.

## Introduction

Cyclophosphamide (CP) is a chemotherapy that is broadly prescribed for the treatment of different types of cancer such as lymphomas, leukemia and multiple myeloma^1^. CP may be also used in treatment of several autoimmune diseases such as rheumatoid arthritis, multiple sclerosis and systemic lupus erythematosus^2^. CP causes serious adverse effects that can result in multiple organs damage. Among these adverse effects are cardiotoxicity, hepatotoxicity, nephrotoxicity and lung toxicity^3,4^. CP is primarily metabolized to the active metabolite 4-hydroxycyclophosphamide which is equilibrates with its tautomer; aldophosphamide. The freely diffusible proportion of aldophosphamide into cells is minor, which it is subject to decomposition into two phosphoramide mustard and acrolein^5^. Phosphoramide mustard is responsible for the antineoplastic effect of CP, while its adverse effects, are mainly caused by acrolein^6^.

Previous studies reported that the pathophysiology of CP toxicity is due to the induced oxidative stress where acrolein perturbs the cellular antioxidant system by generating reactive oxygen species (ROS).The oxidative stress results in increased production of pro-inflammatory cytokines and stimulation of several signaling pathways and thus leading to inflammation, fibrosis and apoptosis^3,4^.

Nrf-2 activates the antioxidant response element-(ARE-) induced cytoprotective gene transcription and is therefore considered a key transcription factor that functions to combat inflammation and oxidative stress. The genes regulated by the Nrf2/ARE pathway are more than 500 genes, having a those that control inflammation (TGF-*β* and NF-*κ*B), oxidative stress such as (HO-1, GCLM, and GCLC), apoptosis (Bcl-2 and Bcl-xL), autophagy (p62) as well as xenobiotic metabolism and excretion (NQO1, AKR1C1, and MRP1)^7^. Moreover, in order for the protective effects of Nrf-2 to function, its activation should be strictly regulated. Previous studies reported that Nrf-2 is relatively abundant in tissues where cascades of antioxidation and detoxification are routinely processed, such as the kidneys, lungs and liver. Moreover, the Nrf-2 expression levels showed significant correlation with the vulnerability, severity, and healing of airway disorders^7^. Several mechanisms are involved in the Nrf-2 regulation including canonical (Keap 1 dependent) mechanism that activates the Nrf-2 signaling and non-canonical (Keap1-independent) mechanisms which can regulate Nrf-2 either positively or negatively. Among these non-canonical mechanisms are the protein kinases which play an essential role in the regulation of Nrf-2. Among the various representative protein kinase pathways are MAPKs, phosphatidylinositol 3-kinase (PI3K), PKC, and glycogen synthase kinase-3 (GSK-3). The regulatory role of the latter kinases is either stimulatory or inhibitory. The positive regulation of Nrf2 is ensued via its phosphorylation by PI3K, PKC, c-Jun, N-terminal kinase (JNK) and extracellular signal-regulated protein kinase (ERK), while the bi-faceted regulation of Nrf2 pathway by p38 MAPK is ensued both positively and negatively. Recently, another noncanonical pathway of Nrf2 activation involving autophagy is reported to be closely associated with the autophagy substrate p62. However, autophagy-mediated Nrf2 activation exerts both positive and negative functions; where the induction of autophagy leads to controlled Nrf2 activation, exerting thereby protective effects. Conversely, the dysregulation of autophagy results in excessive Nrf2 activation that leads to pathological negative effects. Such findings drew the attention towards the investigation of potential Nrf-2 activators^7^.

Among the signaling pathways activated by CP induced oxidative stress are the nuclear factor kappa B (NF-κB) pathway, and the mitogen activated protein kinases (MAPK) pathway. The increase in the concentration of proinflammatory cytokines like IL-6 and TNF-α results in the activation of NF-κB leading to increased cell growth and proliferation as well as increased transcription of the proapoptotic genes and eventually causing apoptotic cell death^8^. In addition, oxidative stress results in the activation of several MAPK regulators such as p38 and ERK1/2 resulting in further activation of NF-κB and the increase in the cell proliferation^9^.

Sacubutril/valsartan, a first-in-class neprilysin inhibitor/angiotensin II AT1-receptor, has a unique mode of action that targets both the RAAS system and the natriuretic peptides pathways. It functions via blocking the AT1 receptor, resulting in the inhibition of the RAAS system, while inhibition of the neprilysin enzyme prevents the degradation of the natriuretic peptides, causing elevation of their cellular levels^10^. Brain natriuretic peptide (BNP), a family member of the natriuretic peptides (NPs), has been demonstrated to be involved in several biological processes mainly in the cardiovascular system, such as regulating heart and renal homeostasis, as well as modulating vascular cell functions^11^. Several studies focused on the regulatory effect of BNP on the inflammatory mediators and showed that recombinant BNP (rhBNP) inhibited the oxidative stress and regulated the expression of the inflammatory cytokines IL-6 and TNF-α through inhibiting NF-κB and MAPK pathways^12,13^. This prompted us to investigate the potential protective role of sacubitril/valsartan against CP induced lung injury via BNP dependent pathway.

Recently, growing evidences imply that oxidative stress induces a series of small noncoding RNAs (microRNA). One of this series is miR-150-3p which is expressed in different organs including the lungs. The role of miR-150-3p in oxidative stress is controversial where, recent studies showed that miR-150-3p was triggered by oxidative stress and was not dependent on p53 overexpression^14^. However, other studies showed that miR-150-3p was downregulated by oxidative stress and that it has a cytoprotective action by targeting p53^15^. It was also reported that miR-150-3p could be stimulated by NF-κB activation^14^. These results drew our attention to the importance of investigating the underlying interaction between BNP, NF-κB and miR-150-3p, as well as the molecular mechanisms underlying the oxidative stress response in CP induced lung injury.

## Results

### The influence of sacubitril/valsartan and valsartan on the antioxidant Nrf-2 gene expression and protein level in lung tissue homogenate

**Figure 1:**
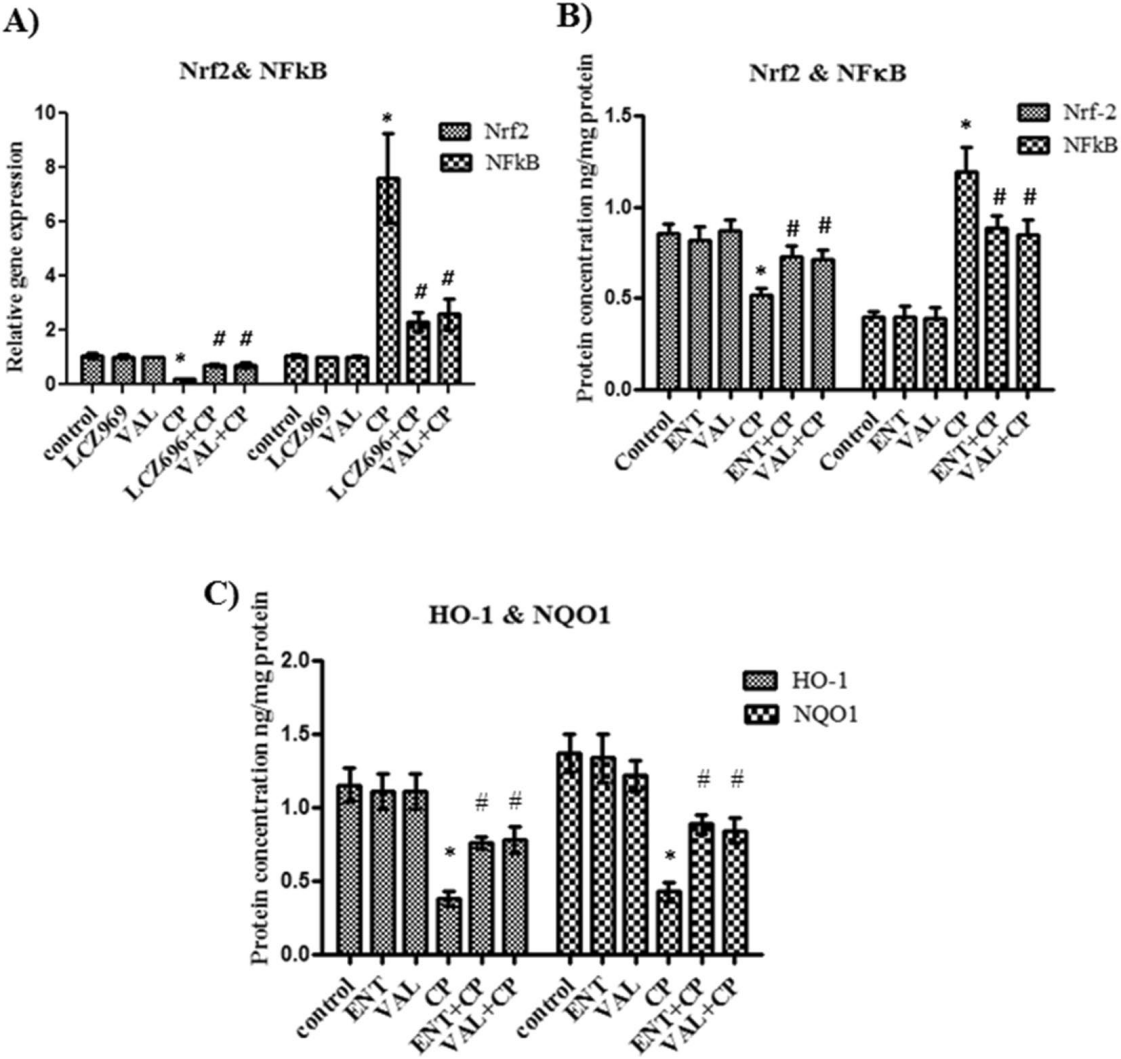
The effect of pretreatment with ENT (30 mg/kg; p.o.) for 6 days, and VAL (15 mg/kg; p.o.) for 6 days, in CP-treated rats (200 mg/kg; i.p.) single dose on day 5, on the antioxidant Nrf-2, NFκB genes expression (**A**) and proteins concentrations (**B**) HO-1 and NQO1 proteins levels (**C**). ENT (sacubitril/valsartan), CP (cyclophosphamide), VAL (valsartan). Data are expressed as mean ± SD (n = 6). Multiple comparisons were performed using one-way ANOVA followed by Tukey’s as post hoc test; *P < 0.05, versus the control group; ^#^*P* < 0.05, versus the CP-treated group.

To study the effect of sacubitril/valsartan and valsartan on the antioxidant status in lung tissues, the relative gene expression and protein level of the antioxidant protein Nrf-2 was measured using quantitative real time PCR and ELISA, respectively. Normal rats treated with sacubitril/valsartan or valsartan showed no significant difference in the relative gene expression or the protein concentration of Nrf-2 compared to control rats (Fig. 1A,B respectively). In CP treated rats, both the relative gene expression and the protein level of Nrf-2 was significantly decreased compared to control rats (Fig. 1A,B respectively). Pretreatment with sacubitril/valsartan or valsartan followed by CP showed a marked increase in the relative gene expression of Nrf-2 together with an almost 40% increase in the protein concentration of Nrf-2 compared to CP treated rats (Fig. 1A,B respectively). However, there was no significant difference between the relative gene expression of Nrf-2 or the protein level following treatment of the rats with either sacubitril/valsartan or valsartan (Fig. 1A,B respectively).

### The influence of sacubitril/valsartan and valsartan on the hemeoxygenase-1 (HO-1) and NAD(P)H quinone dehydrogenase 1 (NQO1) proteins levels in lung tissue homogenate

In order to confirm the effect of sacubitril/valsartan and valsartan on the antioxidant system in the CP induced lung injury, ELISA was used for the quantitative determination of the proteins levels of HO-1 and NQO1 antioxidant enzymes which are the downstream antioxidant enzymes of Nrf2. Treatment of normal rats with each of sacubitril/valsartan or valsartan showed no significant change in the proteins levels of both HO-1and NQO1 compared to control rats (Fig. 1C). In the rats treated with CP alone, both HO-1 and NQO1 proteins levels were markedly decreased to approximately one third of their values in the control rats (Fig. 1C). Moreover, the combination of CP with sacubitril/valsartan or valsartan caused almost a twofold increase in the proteins levels of both HO-1 and NQO1 compared to CP single treatment (Fig. 1C). However, for both enzymes, there was no significant difference between their proteins levels following the combination with sacubitril/valsartan or valsartan (Fig. 1C).

### The effect of sacubitril/valsartan and valsartan on the NF-κB gene expression and protein level in lung tissue homogenate

For further explanation of the mechanism of protection of sacubitril/valsartan and valsartan against CP induced lung injury the relative gene expression of NF-κB was assessed in the lung tissues using quantitative real time PCR and the protein expression of NF-κB was assessed by ELISA. Treatment of normal rats with each of sacubitril/valsartan or valsartan showed no significant change in the relative gene expression or the protein level of NF-κB compared to control rats (Fig. 1A,B respectively). Rats treated with CP only showed about eightfold increase in the gene expression and threefold increase in the protein level of NF-κB compared to control rats (Fig. 1A,B respectively). While, combination of CP with sacubitril/valsartan or valsartan caused almost 70% and 65% decrease in the relative gene expression of NF-κB, respectively with a consequent decrease in the protein levels by about 74% and 71%, respectively (Fig. 1A,B respectively). On the other hand, there was no significant difference between the relative gene expression or the protein levels of NF-κB in both combinations (Fig. 1A,B respectively).

### The influence of sacubitril/valsartan and valsartan on the miR-150-3p gene expression in lung tissue homogenate

**Figure 2:**
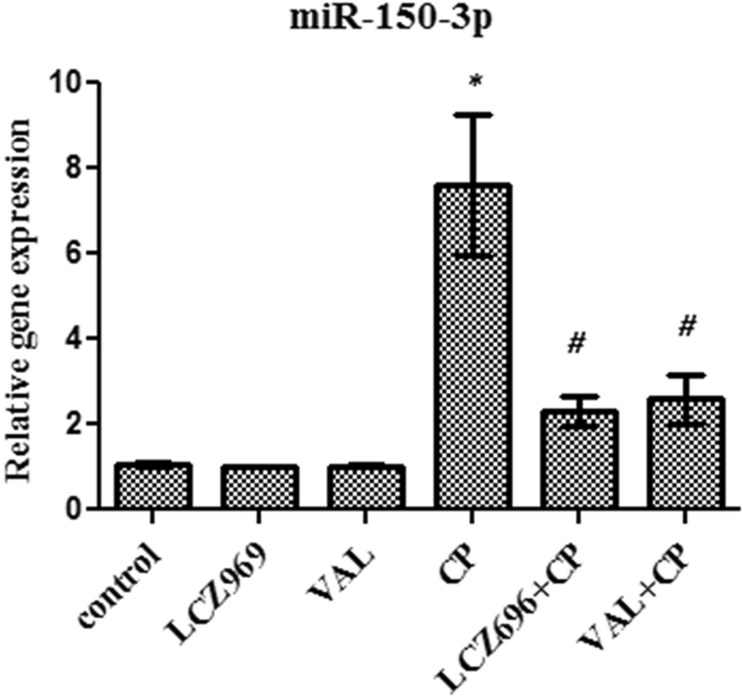
The effect of pretreatment with ENT (30 mg/kg; p.o.) for 6 days, and VAL (15 mg/kg; p.o.) for 6 days, in CP-treated rats (200 mg/kg; i.p.) single dose on day 5, on the relative gene expression of miR-150-3p. ENT (sacubitril/valsartan), CP (cyclophosphamide), VAL (valsartan). Data are expressed as mean ± SD (n = 6). Multiple comparisons were performed using one-way ANOVA followed by Tukey’s as post hoc test; *P < 0.05, versus the control group; ^#^*P* < 0.05, versus the CP-treated group.

To investigate the mechanism by which sacubitril/valsartan and valsartan protects against cyclophosphamide induced lung injury, the relative gene expression of miR-150-3p was determined in the lung tissue using quantitative real time PCR. Both sacubitril/valsartan and valsartan produced no significant difference in the miR-150-3p gene expression compared to control rats. Single treatment with CP caused a significant increase in the relative gene expression of miR-150-3p compared to control rats. Both combinations markedly decreased the relative gene expression of miR-150-3p compared to single CP treatment. However, no significant difference was observed in the gene expression of miR-150-3p in both combinations (Fig. 2).

### The effect of sacubitril/valsartan and valsartan on the p-p38 and p-ERK1/2 MAPKs in lung tissue homogenate

**Figure 3:**
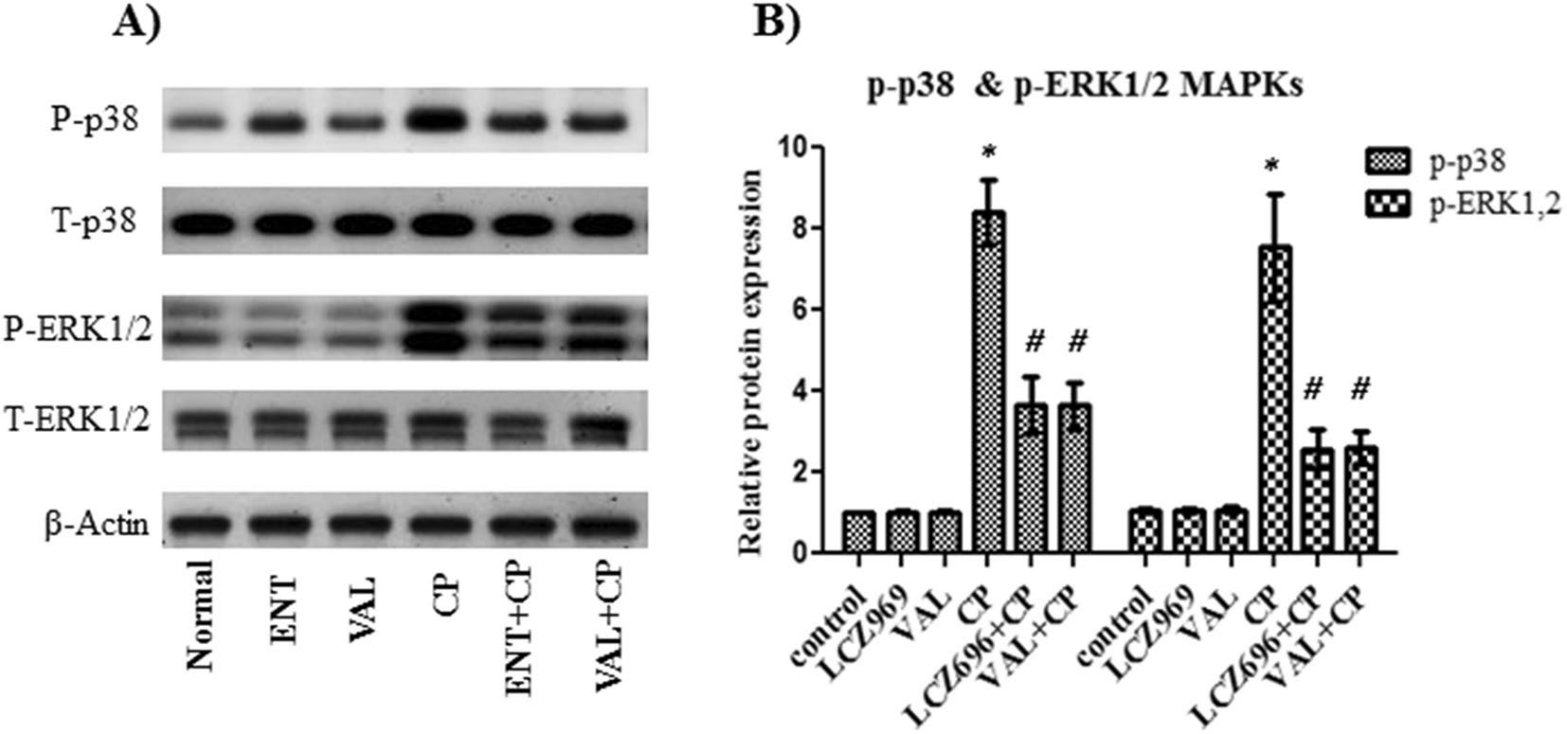
The effect of pretreatment with ENT (30 mg/kg; p.o.) for 6 days, and VAL (15 mg/kg; p.o.) for 6 days, in CP-treated rats (200 mg/kg; i.p.) single dose on day 5, on p38 and ERK1/2 MAPKs, (**A**): the cropped pattern of the protein expression of p38 and ERK1/2 MAPKs, (**B**): the relative proteins expression. ENT (sacubitril/valsartan), CP (cyclophosphamide), VAL (valsartan). Data are expressed as mean ± SD (n = 6). Multiple comparisons were performed using one-way ANOVA followed by Tukey’s as post hoc test; *P < 0.05, versus the control group; ^#^*P* < 0.05, versus the CP-treated group. The full-length blots are presented in the supplementary figure.

In order to explain the mechanism of protection of sacubitril/valsartan and valsartan against CP induced lung injury, the relative protein expression of p38, MAPK, ERK1/2 and MAPK were detected using western blot. Treatment of normal rats with either sacubitril/valsartan or valsartan did not show any significant change in the relative proteins expression of p38 and ERK1/2, compared to control rats (Fig. 3). In CP treated rats the relative proteins expression of p38 and ERK1/2 were significantly increased by about eightfolds compared to control rats (Fig. 3). Both combinations with CP caused about a 40% decrease in the relative expression of p38 and a 50% decrease in the relative expression of ERK1/2 compared to single treatment with CP (Fig. 3). However, no significant difference was observed between the relative expression of p38 or ERK1/2 in both combinations (Fig. 3).

### The effect of sacubitril/valsartan and valsartan on the total and differential leucocytic cells count in BALF

To assess the influence of sacubitril/valsartan and valsartan on the accumulation of the inflammatory cells, the total and differential leucocytic cells count were measured in the BALF pellet using the hemocytometer. Treatment of normal rats with sacubitril/valsartan or valsartan did not show any significant change in the total or the differential leucocytic cells counts compared to control rats (Table 1). Single treatment with CP produced a threefold increase in the total leucocytic cells count compared to control rats. Moreover, significant changes were also observed in the differential cells counts, where a 60% decrease in the percentage of lymphocytes and a 50% decrease in the percentage of macrophages together with a 15 fold increase in the percentage of neutrophils and a tenfold increase in the percentage of eosinophils were observed in CP treated rats compared to control rats (Table 1). Pretreatment with sacubitril/valsartan or valsartan followed by CP showed a marked decrease in the total leucocytic cells count compared to single CP treatment, while in comparison to control rats there was no significant difference in the total leucocytic cells counts following each of the pretreatments (Table 1). A significant decrease in the percentages of neutrophils and eosinophils was observed while the percentages of lymphocytes and macrophages was markedly increased by both pretreatments compared to CP treated rats (Table 1).

**Table 1:** The total and differential leucocytic counts in the BALF pellets of the control and all experimental groups.

Groups	Total cells count × 10^5^	Lymphocytes %	Neutrophils %	Macrophages %	Eosinophils %
Control	4.48 ± 0.33	13.25 ± 0.88	2.92 ± 0.37	83.17 ± 6.9	0.55 ± 0.05
ENT	4.46 ± 0.46	11.88 ± 2.02	2.87 ± 0.12	84.63 ± 3.59	0.49 ± 0.09
VAL	4.46 ± 0.49	11.6 ± 1.93	2.9 ± 0.44	84.11 ± 5.61	0.51 ± 0.08
CP	12.8 ± 0.7*	5.47 ± 1.29*	45.3 ± 3.08*	43.2 ± 6.49*	5.15 ± 0.65*
ENT + CP	4.8 ± 0.49^#^	9.63 ± 0.59*^#^	10.9 ± 1.12*^#^	77.9 ± 6.77^#^	1.22 ± 0.16^#^
VAL + CP	5.85 ± 0.96^#^	9.41 ± 0.96*^#^	13.63 ± 2.32*^#^	74.5 ± 7.48^#^	1.84 ± 0.27*^#^

Data are presented as the mean ± SD (One-way ANOVA followed by Tukey’s multiple comparison test; *P < 0.05, versus the control group; ^#^*P* < 0.05, versus the CP-treated group).

### The effect of sacubitril/valsartan and valsartan on the total protein content level and LDH activity in BALF

**Figure 4:**
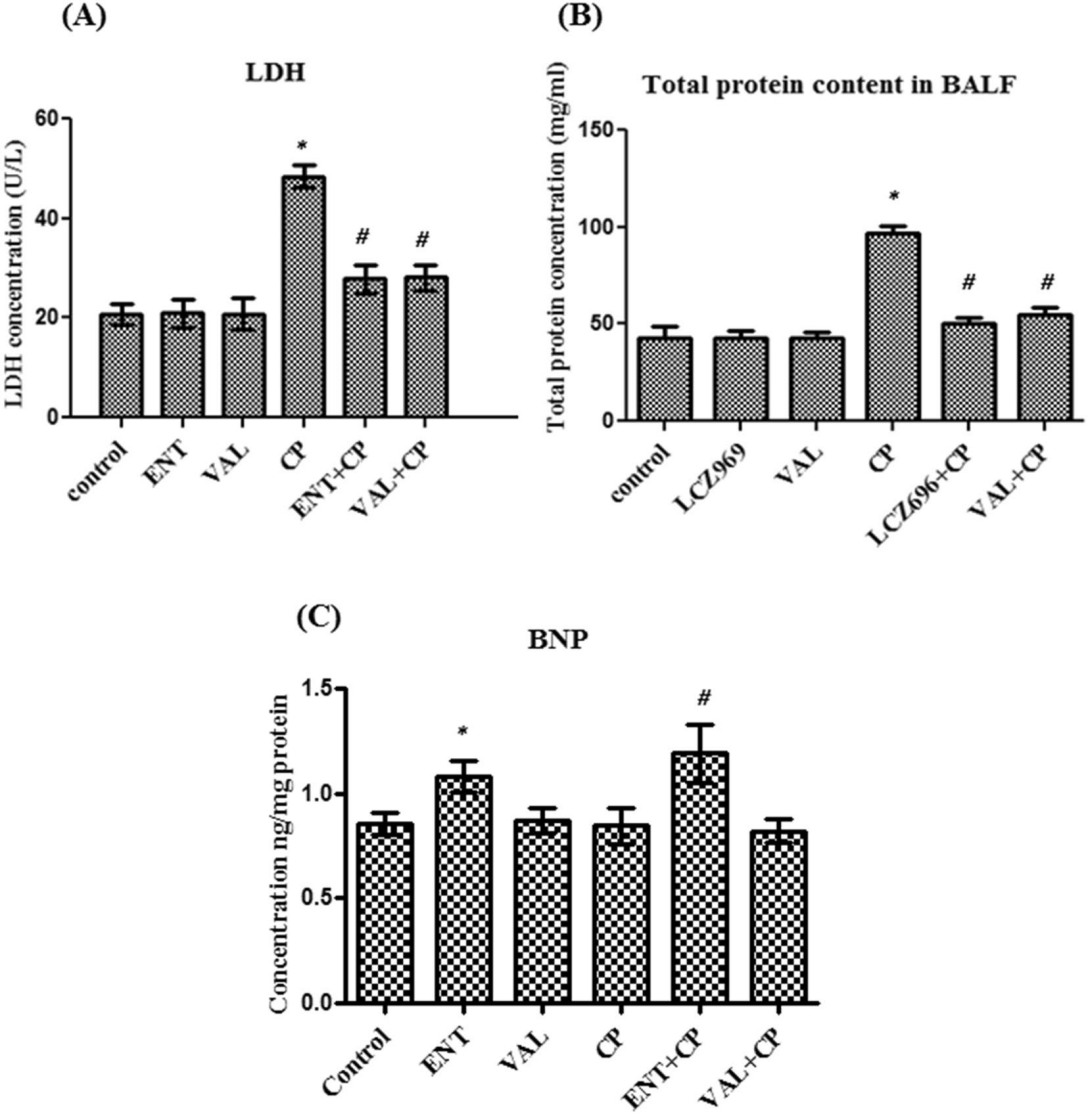
The effect of pretreatment with ENT (30 mg/kg; p.o.) for 6 days, and VAL (15 mg/kg; p.o.) for 6 days, in CP-treated rats (200 mg/kg; i.p.) single dose on day 5, on (**A**) the LDH activity and (**B**) the total protein content and (**C**) the BNP concentration in BALF. ENT (sacubitril/valsartan), CP (cyclophosphamide), VAL (valsartan). Data are expressed as mean ± SD (n = 6). Multiple comparisons were performed using one-way ANOVA followed by Tukey’s as post hoc test; ^*^P < 0.05, versus the control group; ^#^*P* < 0.05, versus the CP-treated group.

To determine the effect of sacubitril/valsartan and valsartan on the epithelial and cell membrane integrity, the total protein content in BALF was measured according to the method of Smith et al. (1985). In addition, the LDH activity in BALF was measured colorimetrically. Treatment of normal rats with either sacubitril/valsartan or valsartan did not produce any significant change in the LDH activity or the total protein content compared to control rats (Fig. 4A,B). Single treatment with CP produced about two fold increase in both parameters compared to control rats while pretreatment with sacubitril/valsartan followed by CP caused about a 40% decrease in LDH and an approximately 50% decrease in the total protein content compared to single CP treatment (Fig. 4A,B). Combination of valsartan and CP caused approximately 40% decrease in both parameters compared to single CP treatment (Fig. 4A,B). However, there was no significant difference between the effect of both combinations on both parameters (Fig. 4A,B).

### The influence of sacubitril/valsartan and valsartan on the Brain natriuretic peptide (BNP) in BALF

To confirm the effect of sacubitril/valsartan and valsartan on the BNP system in the lung, the BNP protein level was measured in the BALF using ELISA. There was no apparent change in the protein levels of BNP in all normal rats receiving either valsartan or CP alone compared to control rats (Fig. 4C). In addition, pretreatment with valsartan followed by CP did not cause any change in the BNP protein level compared to single CP treatment (Fig. 4C). However, treatment of normal rats with sacubitril/valsartan caused about 37% increase in the BNP protein level compared to control rats while, pretreatment with sacubitril/valsartan followed by CP caused almost 50% increase in the BNP protein concentration compared to single treatment with CP (Fig. 4C).

### The influence of sacubitril/valsartan and valsartan on the pro-inflammatory cytokines in BALF

**Figure 5:**
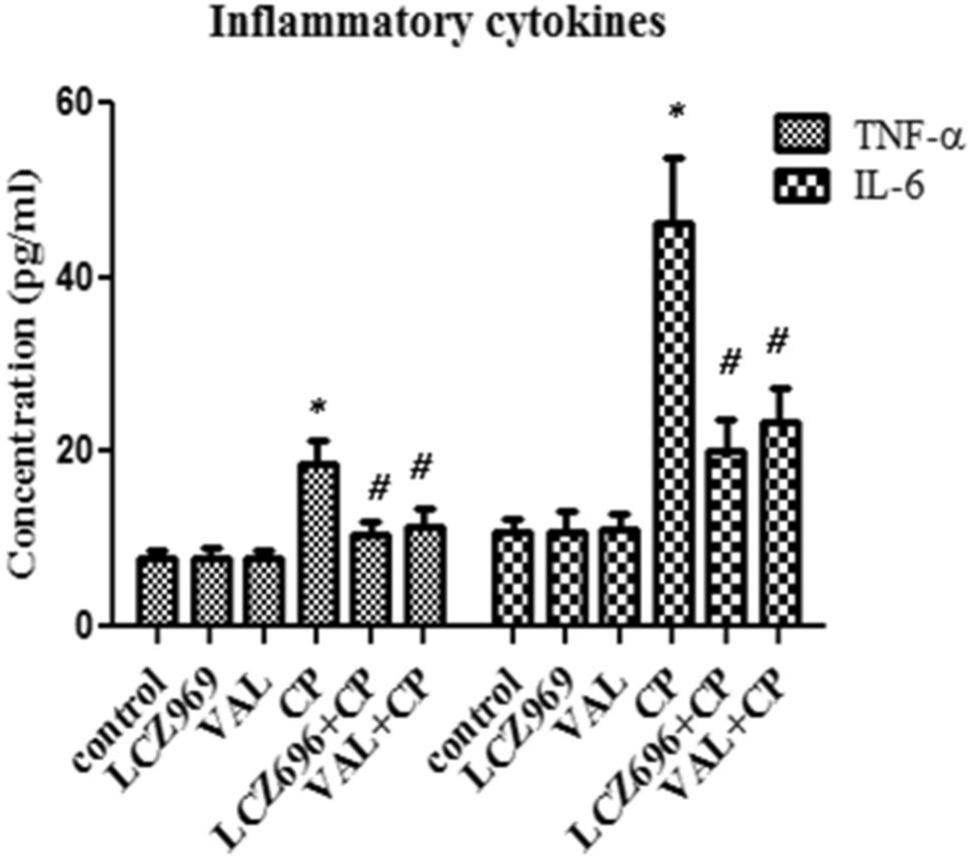
The effect of pretreatment with ENT (30 mg/kg; p.o.) for 6 days, and VAL (15 mg/kg; p.o.) for 6 days, in CP-treated rats (200 mg/kg; i.p.) single dose on day 5, on the pro-inflammatory cytokines in BALF. ENT (sacubitril/valsartan), CP (cyclophosphamide), VAL (valsartan). Data are expressed as mean ± SD (n = 6). Multiple comparisons were performed using one-way ANOVA followed by Tukey’s as post hoc test; ^*^P < 0.05, versus the control group; ^#^*P* < 0.05, versus the CP-treated group.

To assess the influence of sacubitril/valsartan and valsartan on the proinflammatory cytokines, the level of IL-6 and TNF-α were measured using ELISA technique. Both sacubitril/valsartan and valsartan treatment in normal did not produce any apparent change in both parameters compared to control rats (Fig. 5). Single CP treatment produced about two folds increase in the level of TNF-α and four folds increase in the level of IL-6 compared to control rats (Fig. 5). Both Combinations with cyclophosphamide caused about 40% decrease in the level of TNF-α and a 50% decrease in the level of IL-6 compared to single CP treatment (Fig. 5). There was no apparent difference between the levels of both parameters in both combinations (Fig. 5).

### Histopathological and immunohistochemical results

#### Light microscopic results

**Figure 6:**
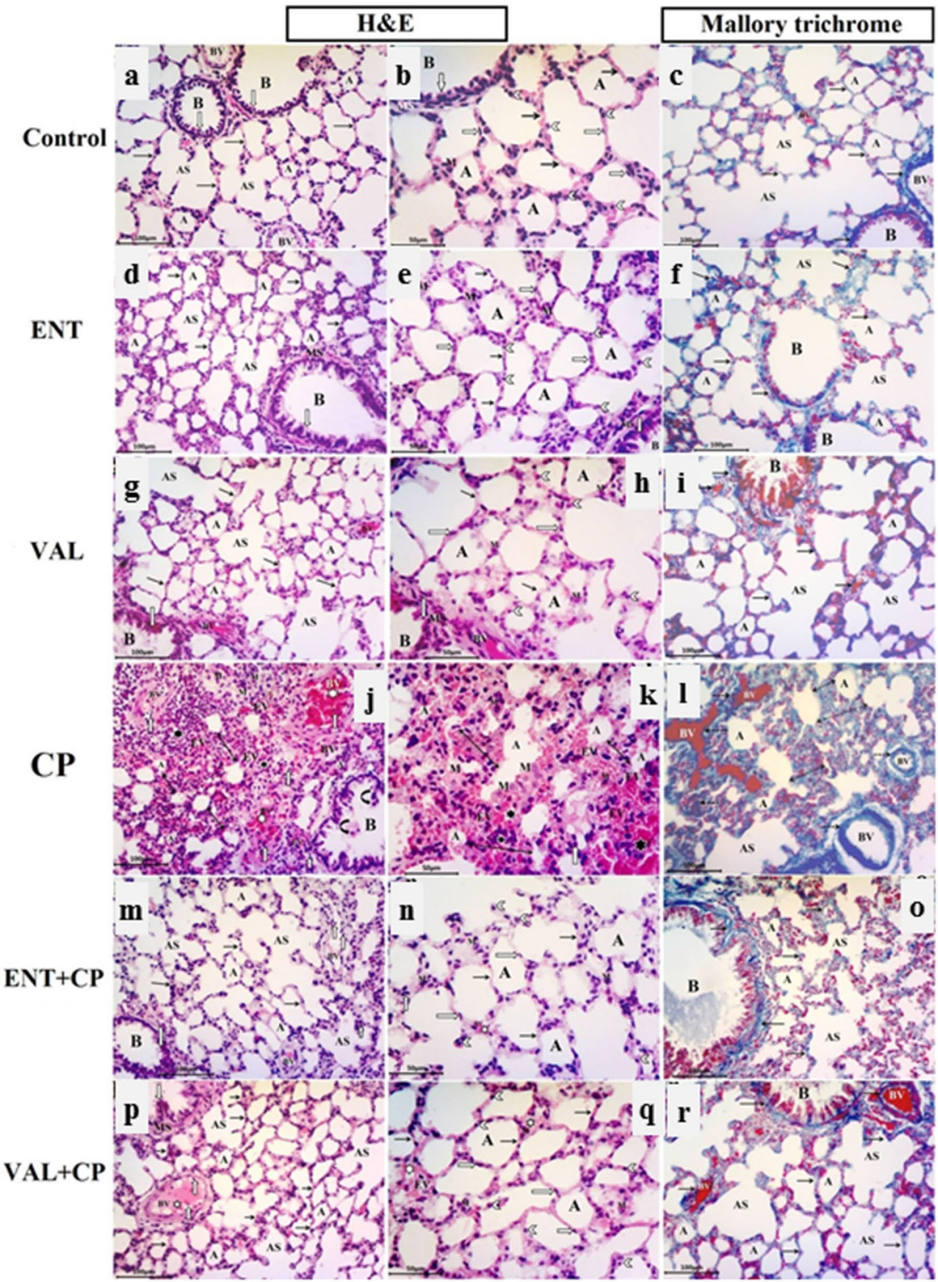
Photomicrographs of lung sections from all experimental groups. ENT (sacubitril/valsartan), CP (cyclophosphamide), VAL (valsartan). Control group (**a**–**c**): (**a**) normal alveoli (A) separated by thin interalveolar septa (black arrow), alveolar sacs (AS), A patent bronchiole (B) , pulmonary blood vessels (BV). (**b**) Squamous type I pneumocyte with flattened nuclei (transverse white arrow) and cuboidal type II pneumocytes with rounded nuclei (white arrow head) bulging into the alveolar lumen. A few macrophages (M) in the interalveolar septa. Bronchiole (B) with simple columnar epithelium (vertical white arrow) and smooth muscle layer (SM). (**c**) Fine collagen fiber in alveolar sacs (AS) and in the thin interalveolar septa (black arrow) in between alveoli (A), around bronchiole (B) and blood vessels (BV). ENT and VAL groups (**d**–**i**): The normal alveolar architecture and collagen distribution appear as the control. CP-induced group (200 mg/kg; i.p.) single dose on day 5 (**j**–**l**): (**j**, **k**) narrowed alveolar spaces (A), thick interalveolar septa (double head arrow) , diffuse lymphocyte infiltration (black star), extravasated RBCs (EV), thick congested pulmonary blood vessels (white star) with lymphocytic infiltration (white arrow). Desquamated cells (curved arrows) in the bronchiolar lumen (B). (**k**) Large alveolar macrophages (M) with vaculated acidophilic cytoplasm in the pulmonary interstitium and within alveolar lumen. (**l**): Excessive collagen fiber deposition in thick interalveolar septa (detached double head arrow) and around congested pulmonary blood vessel (detached arrows). ENT + CP (30 mg/kg; p.o. for 6 days and 200 mg/kg; i.p. single dose on day 5, respectively) and VAL + CP groups (15 mg/kg; p.o. for 6 days and 200 mg/kg; i.p. single dose on day 5, respectively) (**m**–**r**): show a potentially alleviated lung architecture. Thickening of interalveolar septa in some regions (black arrow) with congested intervening blood vessels (white star) and a few extravasated RBCs (EV). (**o**, **r**) Normal distribution of collagen fibers in the relatively thin interalveolar septa (arrows), in between alveoli (A) , alveolar sacs (AS) , around bronchioles (B) and pulmonary blood vessels (BV) (H & E stain, X200, X400) and (Mallory trichrome stain, X200).

Light microscopic examination of sections of adult rat lung from the control group (Fig. 6a–c) revealed normal spongy histological architecture of the lung tissue with variable sized polygonal clear patent alveoli separated by thin interalveolar septa, alveolar sacs, a patent bronchiole of different sizes intermixed with the alveoli and lined with simple columnar epithelium, surrounded by smooth muscle and connective tissue lamina. The alveoli and alveolar sacs were mostly lined by squamous type I pneumocytes with flattened nuclei and a few cuboidal type II pneumocytes with rounded nuclei bulging into the alveolar lumen. A few alveolar macrophages and some interstitial cells appeared in the thin interalveolar septa as seen by H&E stain. In Mallory trichrome stained sections, collagen fibers were normally distributed as fine fibers within the lung interstitium, thin interalveolar septa, around the wall of pulmonary blood capillaries and bronchioles.

Lung sections in the sacubitril/valsartan and the valsartan groups revealed a high similarity to the normal alveolar pattern and normal distribution of collagen fibers within lung interstitium tissues and in thin interalveolar septa in comparison to the control group (Fig. 6d–i).

Evident exacerbated histological alterations were observed in the lungs of CP induced rats (Fig. 6j–l), in the form of marked thickening of the interalveolar septa, along with numerous diffuse lymphocytic inflammatory cellular infiltration in the lung interstitium andconnective tissue lamina of bronchiole, extravasated red blood cells within the lung interstitium, the thich inter-alveolar septa and in the alveolar lumen. Many areas revealed narrowed and collapsed alveoli, thick congested inter-alveolar pulmonary blood vessels infiltrated with inflammatory lymphocytes. Moreover, many large alveolar macrophages with vaculated acidophilic cytoplasm were seen in the pulmonary interstitium and within alveolar lumen. Desquamated cellular debrisin the bronchiolar lumen was also noticed by H&E stain. Extensive interstitial fibrosis in the form of excessive collagen fiber deposition in the interstitium, in the thick interalveolar septa and around congested blood vessels as well could be detected with Mallory trichrome stain.

Sacubitril/valsartan and valsartan pre-treated CP-induced rats (Fig. 6m–r) revealed significant ameliorated changes in histopathological results of the lung parenchyma that appeared more or less similar to that of the control, in spite of mild thickening in some regions of interalveolar septa with congested blood vessels and a few extravasated RBCs. Normal condensation and distribution of collagen in the relatively thin interalveolar septa, around bronchioles and pulmonary blood vessels was seen with Mallory trichrome stain.

#### Histochemical and immunohistochemical results

**Figure 7:**
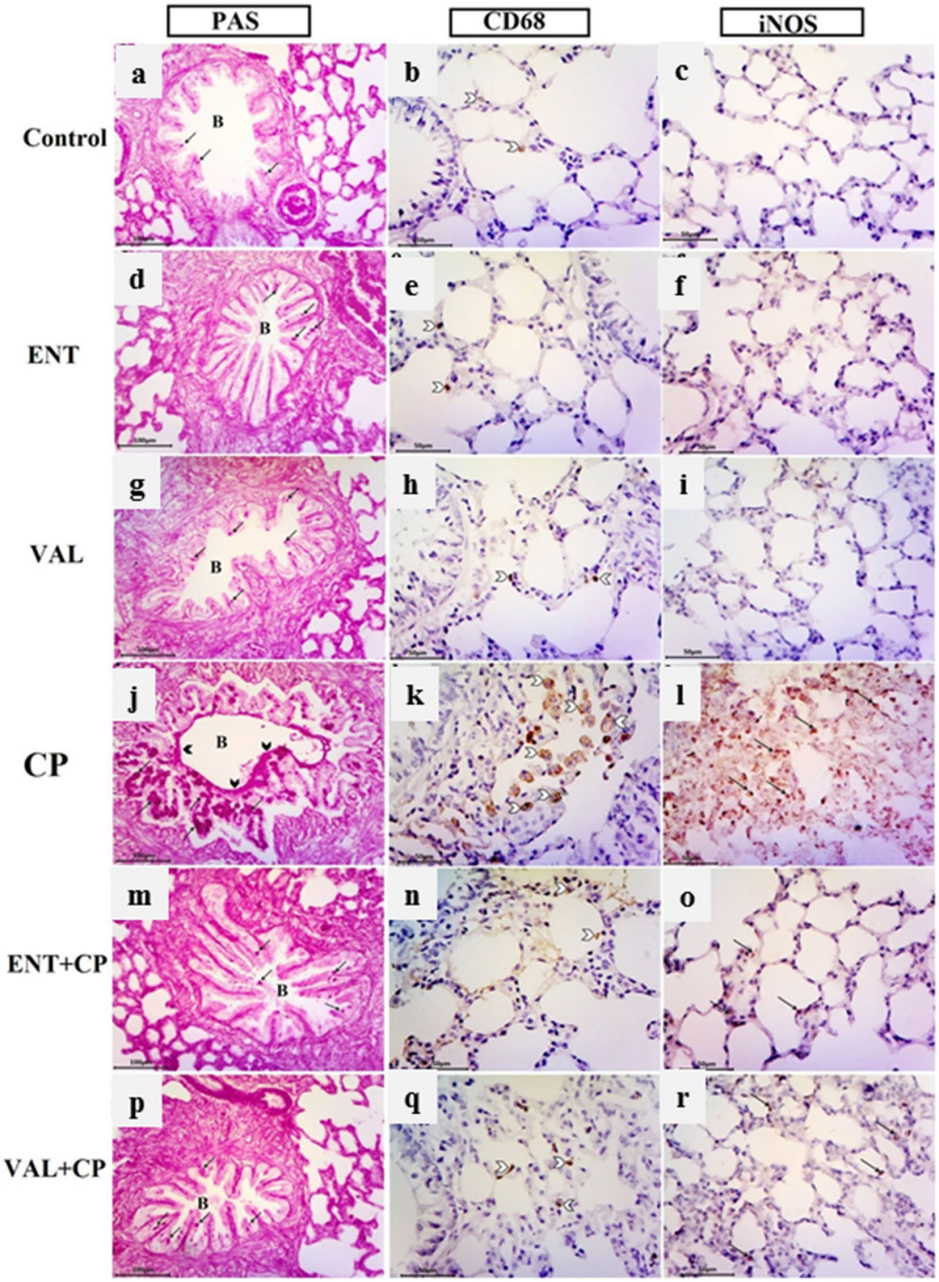
Photomicrographs of PAS reaction, alveolar macrophages CD68 and iNOS immunohistochemistry in the different groups. ENT (sacubitril/valsartan), CP (cyclophosphamide), VAL (valsartan). Control group (**a**–**c**): (**a**) Normal pattern of PAS-positive reaction in a few goblet cells (arrows) of the bronchialepithelial lining. (**b**) A few brown positively staining CD68 alveolar macrophages (white arrow head). (**c**) Undetectable iNOS expression in the alveolar region. ENT and VAL groups (**d**–**i**): similar results to the control are noticed .CP-induced rats (200 mg/kg; i.p. single dose on day 5) (**j**–**l**): (**j**) strong PAS reaction in the numerous goblet cells (arrows) of the epithelial lining of bronchi (B). Mucus can be seen clinging to the apical surface of the epithelial lining (black arrow head). (**k**) Marked dark brown positively stained CD68 alveolar macrophages within the interalveolar septa and in the lumen of alveoli (white arrow head). (**l**) Marked dark brown iNOS immunoreactive cells (arrows) within the interalveolar septa. ENT + CP (30 mg/kg; p.o. for 6 days and 200 mg/kg; i.p. single dose on day 5, respectively) and VAL + CP groups (15 mg/kg; p.o. for 6 days and 200 mg/kg; i.p. single dose on day 5, respectively) (**m**–**r**): (**m**, **p**) Nearly normal pattern of PAS-positive goblet cells (arrows) in the lungbronchi. (**n**, **q**) Decrease of brown positive stained CD68 alveolar macrophages (white arrow head). (**o**, **r**) A few iNOS immunopositive cells within the alveolar and interalveolar tissues (arrows) (PAS technique, X200, alveolar macrophages CD68, X400 & iNOS immunohistochemical staining, X400).

In Fig. 7, PAS reaction for detection of goblet cells inside the bronchi, alveolar macrophages CD68 and iNOS immunohistochemistry were assessed in rat groups. Lung specimen in the control group, stained with PSA technique, revealed normal reaction of only a few goblet cells without any evidence of increased their number (Fig. 7a). Normal distribution of a few brown positively stained CD68 immunoreactive alveolar macrophages was evident in the interalveolar septum of the control group (Fig. 7b).The expression of iNOS immunohistochemistry was undetectable in the alveolar region of lung parenchyma and pneumocytes as well (Fig. 7c). Examination of lung from the sacubitril/valsartan and the valsartan groups gave similar results too (Fig. 7d–i). Conversely, in the CP group, intense strong PAS reaction evident in the numerous goblet cells of the bronchial epithelial lining with markedly increased their number and mucus overproduction clinging to the apical surface of the epithelial lining in comparison to the normal control group (Fig. 7j). Markedly increased dark brown positively stained CD68 immunoreactive alveolar macrophage cells was observed within the interalveolar septa and in the alveolar lumen compared with the control (Fig. 7k). Marked positive dark brown iNOS immunoreactive cells within the interalveolar septa confirming apoptotic changes in lung tissue (Fig. 7l). In the sacubitril/valsartan and valsartan pretreated CP-induced rats, there was normal distribution of PAS positive goblet cells in the lung bronchi without any evidence of increased their number and visible decline in positively stained CD68 alveolar macrophages that appeared in a similar pattern to that in the control group. A noticeable reduction in the expression of iNOS with weakly a few immunopositive cells within the alveolar and inter-alveolar tissues compared with the CP treated group (Fig. 7m–r).

#### Histomorphometrical results

Table 2 reveals significant increase in the mean thickness of interalveolar septa (IAS) [F = 338, *P* ˂ 0.001] by 3,383 folds in H&E stained sections of CP-treated rats, as compared to the control rats. However, a statistically significant decrease in the interalveolar septal thickness upon pre-treatment with each of sacubitril/valsartan and valsartan to 2.13% and 3.6% respectively when compared to the CP-treated group (Table 2). In Mallory trichrome and PAS stained sections, the mean area percentage of collagen fibers at interstitial tissue as well as the percentage of PAS-positive epithelial cells per bronchi were significantly elevated [F = 1,392, *P* ˂ 0.001] and [F = 2096, *P* ˂ 0.001] by 1,643 and 3,347 folds respectively in Cp treated rats, when compared to the control rats (Table 2). Inversely, these characteristics were significantly improved upon pre-treatment with each of sacubitril/valsartan and valsartan, and these percentages reduced to 7.46% and 8.1% of collagen fibers at interstitial tissue, and 3.4% and 5.8% of PAS-positive epithelial cells per bronchi respectively when compared to the CP-treated group (Table 2). Finally, there were significant increase in the mean number of CD68 immunostained alveolar macrophages and in the mean area of the iNOS of CP-treated group [F = 765, *P* ˂ 0.001] and [F = 3,704, *P* ˂ 0.001] by 11% and 3,620 folds, respectively when compared to the control rats (Table 2). Inversely, pre-treatment with sacubitril/valsartan and valsartan significantly declined the CD68 alveolar macrophages to 11.7% and 15.83% and the mean area% iNOS decreased dramatically to 1.44% and 3.04% respectively, in comparison to the CP-treated group (Table 2). Noteworthy, no significant difference was detected between sacubitril/valsartan and valsartan pre-treated groups in all morphometric results (Table 2).

**Table 2:** Histomorphometrical analysis for the lung tissue of the control and all experimental groups.

Parameters		Groups					
		Control	ENT	VAL	CP	ENT + CP	VAL + CP
IAS		0.47 ± 0.4	0.51 ± 0.5	0.53 ± 0.5	15.9 ± 2.3***	0.34 ± 0.4^###^	0.57 ± 0.5^###^
Area % of interstitial collagen		1.99 ± 0.7	2.3 ± 0.52	2.3 ± 0.9	32.7 ± 1.9***	2.5 ± 0.82^###^	2.65 ± 0.8^###^
% of PAS + ve epithelial cells/bronchi		2.02 ± 0.41	2.9 ± 0.54	3.1 ± 0.6	67.6 ± 4.2***	2.7 ± 0.8^###^	3.9 ± 0.9^###^
Mean number ofCD68 alveolar macrophages /HPF		2.2 ± 0.85	2.2 ± 0.9	2.33 ± 0.6	24 ± 1.9***	2.8 ± 0.43^###^	3.8 ± 0.7^###^
Area % of iNOS immunopositivity/HPF		0.01 ± 0.01	0.02 ± 0.01	0.02 ± 0.01	36.2 ± 1.5***	0.52 ± 0.4^###^	1.1 ± 0.4^###^

Data are presented as the mean ± SD (One-way ANOVA followed by Tukey’s multiple comparison test; ***P < 0.001, versus the control group; ^###^*P* < 0.001, versus the CP-treated group).

## Discussion

The therapeutic effectiveness of many anticancer drugs is restricted owing to their marked target organ toxicity and damage^16^, among which cyclophosphamide (CP) is considered among the highly toxic cancer therapeutics. The toxicity of CP, represented on induced cell death, is directly related to the primary toxic metabolite of CP, acrolein, that causes cellular apoptosis, oncosis and necrosis. Acrolein is well known to induce oxidative stress and cause severe, and may be fatal, multiple organ damage^17^. Therefore, there is still an urgent need for potent adjunctive drugs that are able to diminish the toxicity and improve the patients’ quality of life.

Augmentation of natriuretic peptides (NPs) (especially ANP and BNP) proved many beneficial and ameliorative effects in many cardiovascular diseases, owing to its antioxidant, anti-inflammatory properties, as well as antifibrotic impact^18^; however, its potential mechanism in prevention of lung injury remains elusive. Intriguingly, recombinant human brain natriuretic peptide (rhBNP) succeeded to reduce acute lung injury in canine models^19,20^. In addition**,** recent studies showed that brain natriuretic peptides (BNP) might provide a significant protection against target organ damage by inhibition of the oxidative stress^21^. Among the signaling pathways that are activated by the ROS are the Nuclear Factor Kappa B (NF-κB) and the mitogen activated protein kinase (MAPK) pathways that control diverse cellular events such as inflammation, proliferation and apoptosis^22^. In addition, miRNAs are implicated in a plethora of physiological events, including cell proliferation, differentiation, stress condition, and tumorigenesis^23^. Over the past few years, researchers have taken serious attempts in unveiling the controlling impact of miRNAs on the level of expression of natriuretic peptides (NPs) and/or their associated receptors^24^. In this context, we were provoked to investigate the protective effect of sacubitril/valsartan compared to valsartan in a model of CP induced lung injury. To the best of the authors’ knowledge, this is the first study to investigate the possible protective effect of sacubitril/valsartan in CP induced lung injury, and to explore the possible mechanism that might be involved in this protection. Herein, it was focused on exploring the possible involvement of miR-150 in the regulation of NF-κB, MAPK signaling pathways.

Sacubitril/valsartan; a recently drug approved by the FDA, inhibits the degradation of BNP and enhances the beneficial physiological response of NPs while blocking the harmful effects of RAAS activation (tissue damage, inflammation, and fibrosis). Hence, it is not surprising that treatment of CP animals with sacubitril/valsartan, in the current study, significantly ameliorated oxidative stress, inflammation and fibrosis. Rats treated with CP demonstrated a marked decrease in the gene expression and protein level of the antioxidant Nrf-2 with a consequent decrease in the transcriptional downstream antioxidant enzymes HO-1 and NQO1 and a marked augmentation in the percentage area of positive iNOS immunoreactive cells in lung tissue of CP induced rats, compared to control rats. This was consistent with previous studies where, CP induced chronic obstructive pulmonary dysfunction in clinical studies has been associated with oxidative stress and deterioration of the antioxidant defense systems^25^. The CP-induced oxidative stress and consequent generation of ROS cause direct harm to pulmonary vascular endothelial cells. This selective toxicity of CP to pulmonary tissues is augmented by oxidative stress-induced depetion of the detoxifying enzymes, e.g. glutathione, with concurrent increase in lung malondialdehyde (MDA) content^26,27^. The latter causes increased permeability Ca^2+^ across cell membrane, attacks the membrane protein and enzymes, disrupts the capillary membranes of the alveoli, hence allowing proteinaceous fluid to leak into the lung parenchyma causing cellular damage and increasing oxidative stress load^28^. This was also consistent with the current results which showed an apparent increase in the total protein content and the LDH activity in the BALF supernatant, indicating cell injury and loss of the alveolar wall integrity. Fortunately, sacubitril/valsartan caused a significant elevation of the Nrf2 antioxidant gene and protein expression, together with an increase in the proteins expressions of both HO-1 and NQO1, which implies that sacubitril/valsartan might have a protective effect against CP induced lung injury. Similarly, sacubitril/valsartan administration in chronic kidney disease mitigated renal function and histology, and ameliorated most of the molecular markers of oxidative stress, inflammation, fibrosis and increased Nrf2 system. Although, sacubitril/valsartan was more effective than valsartan therapy alone in delaying the progression of kidney disease^29^, however in the current work there was no difference between the results of sacubitril/valsartan and valsartan on Nrf-2 expression in lung tissues.

Reactive oxygen species (ROS) and NF-κB, both have mutual regulatory effects^30^. In the pulmonary tissues, NF-κB regulates ROS via production of pro-inflammatory cytokines including TNF-α , IL-6, and IL-, eliciting an inflammatory cascade and causing neutrophil adherence to pulmonary capillaries, macrophages extravasation into the alveolar space, concomitant with leukocytes sensitizing and infiltration^31,32^. This was confirmed with the current marked elevation in the number of CD68 positive macrophages in pulmonary alveoli in CP induced rats.

The key mechanism underlying the pulmonary airway mucus hypersecretion is the ROS and its induced activation of epidermal growth factor receptor (EGFR). This is ensued via enhancing its kinase activity, receptor modification and structure alteration^33^. The latter activation of EGFR causes also downregulation of Forkhead Box A2 (FOXA2), a known mucin genes transcriptional repressor, although the underlying pathway is yet to be unveiled^34^. Another sequel of oxidative stress-mediated EGFR activation is the over expression of the antiapoptotic protein, Bcl-2^35,36^. Importantly, all three mechanisms resulting in goblet cell hyperplasia in the airway epithelium were associated with increased secretory activity^34^. This was consistent with the current results which revealed that CP caused significant increase in the percentage of PAS-positive epithelial cells /bronchi.

The maintained production of pro-inflammatory cytokines developed a chronic state of indefinite inflammation, proliferation and fibrogenesis responses are, hindering thereby the process of tissue repair^37^. A critical player in the pathogenic cascade of fibrosis is TGF-β, through the stimulation of collagen and elevation of hydroxyproline content. Moreover, the vicious cascade of fibrogenesis is activated via enhanced fibroblast stimulation, synthesis of fibronectin as well as via blocking proteases responsible for lysis the extracellular matrix [49,50]^38^. This was consistent with the overt elevation in the area percentage of collagen at lung interstitium in CP group in the current study.

Several experimental studies were conducted to unveil the interplay between the Nrf2 and NF-κB trajectories, but contradictory outcomes were revealed^39^. In consistence with our results, Nrf2 was reported to impede NF-κB activity, as Nrf2 knockdown experiment caused a marked elevation of the NF-κB transcriptional activity^40^. Furthermore, two of the most critical pathways by Sulforaphane-a potent chemopreventive—in prostate cancer were Nrf-2 activation and NF-κB inhibition^41^. This supports that the antitumor impact displayed by Nrf-2 is achieved by both pathways; activation of antioxidant machinery as well as the inhibitory action of NF-κB mediated pro-inflammatory processes^42^. Accordingly, both sacubitril/valsartan and valsartan caused a significant decrease in the upregulated expression of NF-κB caused by CP in the present study.

In assessment of the inflammatory markers, the current data revealed that CP produced e in the level of TNF-α and IL-6 and both sacubitril/valsartan and valsartan decreased their levels in BALF. This was reflected in the decrease in the total and differential cell count and LDH activity. These effects could be due to the correlation between BNP and inflammatory factors like TNF-α and IL-6. These findings were evidenced by the inhibitory effect of recombinant BNP on the expression of inflammatory factors in vitro and in vivo*.* Additionally, inhibition of these two factors—TNF-α and IL-6—could be through the inhibitory effect of BNP on the p-NF-κB, p-JNK, and p-P38^13^.

For further investigation of the mechanism of protection of sacubitril/valsartan against CP induced lung injury, the proteins levels of P38 and ERK1/2 MAPKs were assessed. A previous investigation highlighted the role of p38-MAPK pathway in the inflammation cascade, by regulating the transcriptional activation of NF-κB, hampering thereby the production of the proinflammatory cytokines^43^. In addition, a previous study showed that in Chinese hamster ovary cells, exposure to acrolein caused cellular apoptosis, which was indeed MAPK-dependent, after activation of the latter by phosphorylation^44^. These results confirmed the implication of MAPKs in acrolein-induced apoptosis which was consistent with the current findings, where CP caused a marked increase in the levels of p38 and ERK1/2 MAPKs. On the other hand, both sacubitril/valsartan and valsartan caused about a 40% decrease in the level of p38 and a 50% decrease in the level of ERK1/2 compared to single treatment with CP. These results suggest that sacubitril/valsartan has a protective effect against lung injury probably due to the inhibitory effect of BNP on the p38 and ERK1/2 MAPKs. Several studies showed similar results where, Iborra-Egea et al. (2017) proved that the ERK signaling pathway was a prospective mechanism of synergism, rationalizing the efficacy of sacubitril/valsartan on cardiac remodeling^45^. Moreover, a recent study showed that the expression of IL-1b was inhibited by BNP through the down-regulation of NF-κB/ERK1/2 and the activation of NALP3/ASC/caspase-1 in humanTHP-1 monocytes^46^.

Previous studies investigated the role of miR-150 in cell survival, apoptosis and inflammation**.** Wan et al., documented that miR-150-3p was one of four miRNAs identified as the oxidative stress-responsive miRNAs in hepatocellular carcinoma^47^. Moreover, Qin et al., showed endothelial apoptosis induced by oxidized low-density lipoprotein (ox-LDL) was accelerated by the ectopic expression of miR-150^48^. In addition, Yang et al., demonstrated that miR-150 suppression had a protective effect against IL-1 injured ATDC5 cells^19^. These previous studies were consistent with the current results that demonstrated that CP caused a significant increase in the relative gene expression of miR-150-3p. On the contrary to our results, Xue et al., showed that the pulmonary inflammation and induced apoptosis could be protected by increased expression of miRNA 150^49^. Moreover, It was projected that the major pro-inflammation signaling pathway, TNF-α/ IKK/NF-kB could directly stimulate miR-150-3p expression via a novel binding site of NF-κB—on the promoter of miR-150^50^. This was consistent with the current results as CP caused significant upregulation of NF-κB expression and subsequently miR-150-3p in lung tissues.

A previous study reported that the propagation and relocation of cancerous pulmonary cells is caused by miR-150 induced repression of kinase signaling inhibitor 1 (SRCIN1), which stimulated the Src/focal adhesion kinase (FAK) and Src/Ras/extracellular signal-regulated kinase (ERK) pathways^51^. This emphasizes the function of miR-150-3p in the induction of CP lung injury, as the current results repoted elevated protein levels of p38 MAPK and ERK1/2 MAPK using western blot technique and showed that the CP treated group displayed the highest levels of p38, ERK1/2 expressions, in comparison to the control group.

Both sacubitril/valsartan and valsartan downregulated the expression of miR-150-3p in lung tissues supporting the previous report which documented that NF-κB activation caused an elevated expression of miR-150-3p, due to the presence of three NF-κB binding sites in the promoter region of miR-150-3p. At the same time, down-regulation of miR-150-3p alleviated inflammatory injury induced by interleukin 1^14^. Moreover, the release of pro-inflammatory cytokines, IL-6 and TNF-α was associated with high expression of miR-150-3p and enhanced activation of NF-κB in human chondrogenic cells^19^. To this end, the current study suggests that the effect of both sacubitril/valsartan and valsartan on these inflammatory factors was most probably due to changes in the levels of miR-150-3p and BNP.

Histopathological findings confirmed the before mentioned biochemical and epigenetic findings and evidenced that sacubitril/valsartan effectively mitigates the vicious CP-induced lung injury. This was affirmed by recruitment of inflammatory cell infiltrations, significant decrease in macrophage number, the area percentage of iNOS expression and collagen fiber deposition at the lung interstitium. These ameliorative events caused improvement in the structure and function of endothelium of the blood vessels which in turn succeeded to subside the CP-induced congestion and extravasation of RBCs. The overall cytoprotective and antioxidant property of sacubitril/valsartan towards the histological architecture of the pulmonary tissue was non-inferior to valsartan.

The findings of the current study sheds light, for the first time, on the therapeutic impact of sacubitril/valsartan against lung toxicity induced by CP in rats. The lung injury, mediated by CP-induced oxidative stress, was associated with miR-150-3p overexpression and consequent inflammatory response, via NF-κB and MAPK pathways. Biochemical, molecular and histopathological data revealed that sacubitril/valsartan exhibited preventive potential against CP-induced oxidative and inflammatory events which was most probably due to the changes in the levels of miRNA 150-3P and BNP level. This was reflected on the oxidative stress and inflammatory markers. Intriguingly, no significant difference between the results of sacubitril/valsartan and valsartan were evident, indicating that the protective impact of sacubitril/valsartan is non-inferior to valsartan. This was most probably due to the short duration of treatment or because the regulatory action of valsartan on the same markers (MAPK and NF-κB) was established through a different pathway.

## Materials and methods

### Chemicals

Sacubitril/valsartan (ENT) (Entresto®) was a kind gift of Novartis Pharma AG (Basel, Switzerland), while valsartan (VAL) was obtained from Sigma-Aldrich Chemical Co. (Missouri, USA). Each of ENT and VAL was formulated as a corn oil emulsion (2 ml/kg; p.o.), and given by oral gastric gavage once daily. Cyclophosphamide (CP) (Endoxan®) was obtained from Baxter AG (Opfikon, Switzerland). CP was diluted with normal saline 0.9% to a final concentration of 2% (20 mg/ml)^53^. All chemicals and reagents, unless otherwise specified, were of highest analytical grade and were acquired from Sigma-Aldrich Chemical Co. (Missouri, USA).

### Animals

Swiss adult albino rats were obtained from the Nile Company for Pharmaceuticals and Chemical Industries (Cairo, Egypt). The total number of rats used was seventy two adult male, with an average body weight of 120 ± 20 g. Prior to experimentation, all rats were divided into three rats per standard polypropylene cage and were allowed an acclimatization period of one-week at the animal facility in the laboratory of Physiology, Faculty of Medicine AI-Azhar University. Throughout the experimental period, all rats were allowed free access to tap water and normal pellet diet (EL Nasr Pharmaceutical Chemicals Co., Cairo, Egypt). The temperature of the animal facility was adjusted to 25 ± 5 °C and relative humidity of 55 ± 5% with 12-light/12-dark cycle^53^.

### Experimental animals and design

Rats were randomly divided into 6 groups, each containing 12 rats as follows:

- *Group 1* Rats received corn oil (2 ml/kg/day; p.o.) for 6 days, to serve as control.
- *Group 2* Rats received corn oil (2 ml/kg/day; p.o.) for 6 days, with CP (200 mg/kg; i.p.) single dose on day 5, to induce lung toxicity^54^.
- *Group 3* Rats received ENT (30 mg/kg; p.o.) for 6 days, to serve as positive control^55^.
- *Group 4* Rats received VAL (15 mg/kg; p.o.) for 6 days, to serve as positive control.
- *Group 5* Rats received ENT (30 mg/kg; p.o.) for 6 days^55^, with CP (200 mg/kg; i.p.) single dose on day 5^54^.
- *Group 6* Rats received VAL (15 mg/kg; p.o.) for 6 days, with CP (200 mg/kg; i.p.) single dose on day 5^54^.

All rats were weighed at the beginning of the experimental protocol, and on day 7 (24 h after the last dose), and weight changes were calculated.

At the end of the experimentation period, six rats from each group were anesthetized with ketamine and subjected to cervical dislocation. Afterwards, both lungs were rapidly separated and washed with ice-cold saline. The right lung was homogenized in isotonic saline to reach 10% homogenate, and the supernatant was saved for biochemical analysis; while the left lungs were rapidly fixed in 10% neutral buffered formalin for 72 h and processed for further light microscopical examination.

Under ketamine anesthesia, the lungs of the other six rats, were dissected from each group to extract the broncho-alveolar lavage fluid (BALF) which was used for assessment of lung injury markers.

### Biochemical analyses of lung tissue homogenates

#### Nrf2 and NF-κB gene expression by quantitative real time PCR

Real-time PCR was performed for quantitative expression of Nrf2 and NF-κB genes. Tissue samples were lysed for total RNA isolation using RNeasy purification reagent (Qiagen, Valencia, CA). Total RNA purity was determined through the absorption ratio (260/280 nm)^56^. It was between 1.8 and 2.0 for all preparations. Reverse transcription of total RNA to cDNA was carried out using reverse transcription reaction (Superscript II, Gibco Life Technologies, Grand Island, NY, USA). Real-time PCR amplification and analysis were done using an Applied Biosystem with software version 3.1 (StepOneTM, USA). Each reaction consisted of 50 ng of cDNA product, 0.2 μM of primers and 10 μl of SYBR® Green Real-Time PCR Master Mix (Applied Biosystems). The reactions were incubated at 95 °C for 3 min, followed by 40 cycles of 95 °C for 15 s, 62 °C for 30 s and 72 °C for 30 s^57^. The mRNA expression of β-actin was considered as the internal control, and all the assessment and analysis of all data was processes using the comparative cycle threshold (CT) method. The primers used for Nrf2 , NF-κB and β-actin were (Nrf2; Forward primer: 5′-GCT GCA GAA GCA AGA GAA CC-3′ and Reverse primer: 5′-GGCAGTGAAGACTGAACTTTCA-3′, GenBank accession number **NM031789.2**^58^, NF-κB; Forward primer: 5′-CATTGAGGTGTATTTCACGG-3′, Reverse primer :5′-GGCAAGTGGCCATTGTGTTC-3′, GenBank accession number **NM199267.2)**^59^, β-actin; Forward primer: 5′-GACGGCCAGGTCATCACTAT-3′, Reverse primer: 5′-CTTCTGCATCCTGTCAGCAA-3′, GenBank accession number **XM011332812.3**)^58^.

#### Detection of miR-150-3p expression level

Total miRNA was extracted from tissues using the mirVana miRNA Isolation Kit (Ambion, Austin, TX, USA) according to the manufacturer’s instructions. Real-time PCR assay was performed to detect the expression of miR-150-3p in tissues. Briefly, 10 µg of Micro RNA was subjected to reverse transcription. The cDNA was amplified through PCR and U6 was used as the endogenous control. The PCR primers used were as follows: miR-150 (GenBank accession number **NR049590.1**) forward, 5-CAG TAT TCT CTC CCA ACC CTT GTA-3 and reverse 5-AAT GGA TGA TCT CGT CAG TCT GTT-3; U6 (GenBank accession number **XR003481952.1**) forward, 5-ATT GGA ACG ATA CAG AGA AGA TT-3 and reverse, 5-GGA ACG CTT CAC GAA TTT G-3^57^. The PCR conditions were: initial denaturation at 95 °C for 3 min, followed by 40 cycles of 95 °C for 15 s, 62 °C for 30 s, and 72 °C for 30 s^57^. Real-time PCR was performed using SYBR Green PCR MasterMix (Applied Biosystems) on an ABI 7300HT real-time PCR system (Applied Biosystems, Foster City, CA, USA). The expression of U6 was used to normalize the relative expression of miR-150-3p. For the purpose of data analysis, the comparative cycle threshold (CT) method was used.

#### Detection of ERK1/2 and P38 by western blot

Briefly, protein extraction from the tissue homogenates was done by adding ice-cold radio immune-precipitation assay (RIPA) buffer containing phosphatase and protease inhibitors (50 mmol/L sodium vanadate, 0.5mMphenylmethylsulphonyl fluoride, 2 mg/mL aprotinin, and 0.5 mg/mL leupeptin) to the tissue homogenates, followed by 20 min centrifugation at 12,000 rpm^60^. The protein assay was performed according to Bradford method. Equal quantities of protein (20–30 µg of total protein) were segregated by SDS/polyacrylamide gel electrophoresis (10% acrylamide gel) using a Bio-Rad Mini-Protean II system and following the instructions manual^60^. The Bio-Rad Trans-Blot system was used transfer the protein to polyvinylidene difluoride membranes (Pierce, Rockford, IL, USA). The membranes were washed with PBS, then, blocked for 1 h at room temperature with 5% (w/v) skimmed milk in PBS^60^. After blocking, the blots were developed using primary antibodies for p- ERK (Cat # 44-680G, 1:1,000) , p-p38 (Cat # 44-684G, 1:1,000) and beta actin (Cat # MA5-15739, 1:1,000) supplied by (Thermoscientific, Rockford, Illinois, USA) incubated overnight at pH 7.6 at 4 °C with gentle shaking. Following washing, secondary antibodies labeled with peroxidase (Cat # PA1-28573, 1:2000) were added and incubated at 37 °C for 1 h^60^. ChemiDocTM imaging system with Image LabTM software version 5.1 (Bio-Rad Laboratories Inc., Hercules, CA, USA) was used to analyze band intensity, after normalization for β-actin protein expression.

#### Detection of Nrf2 and NF-κB proteins levels in the lung tissues homogenates by ELISA

The protein concentration of both Nrf2 and NF-κB were assessed in the lung tissues homogenates of all experimental groups by ELISA using commercial kits purchased from MyBioSource.com (Nrf2: Cat # MBS012148 and NF-κB: Cat # MBS453975). Briefly, for detection of Nrf2 the tissues were homogenized in PBS (10 mg tissue to 100 μl PBS.) then centrifuged at 1,000 × g for 20 min. The supernatant was then collected and assayed according to the manufacturer’s instructions. As for NF-κB detection, tissues homogenization on ice was done in 5–10 mL of PBS. For further shattering of the cell membranes, the obtained suspensions were exposed to two freeze–thaw cycles. Afterwards, the homogenates were centrifuged for 5 min at 5,000× g. and the supernatant were assayed immediately. The optical density was read at 450 nm using an ELISA reader within 15 min for both Nrf2 and NF-κB.

#### Detection of heme oxygenase-1 (HO-1) and NAD(P)H quinone dehydrogenase 1 (NQO1) proteins levels in the lung tissues homogenates by ELISA

The protein levels of the antioxidant enzymes HO-1 and NQO1 were measured in the lung tissue homogenates of all experimental groups using commercial kits purchased from Abcam (HO-1: Cat # ab213968) and LifeSpan Biosciences, Inc. (NQO1: Cat # LS-F32218). The tissues were homogenized, centrifuged and then the supernatants were assayed following the instructions provided by the manufacturer. ELISA reader was used to measure the optical densities at 450 nm.

### Collection of bronchoalveolar lavage fluid (BALF)

To obtain the BALF, a cannula (size: 24G, flow rate: 16 mL/min) was inserted into the exposed trachea after opening of the thoracic cavity. The lung was washed three times. Each time, 3 mL aliquots of saline was injected through the tracheal cannula, kept for 30 s in the lung and then aspirated gently to get a total of 9 mL BALF. The BALF recovery rates were more than 80% with no significant difference among groups. After centrifugation of the collected BALF at 2000 rpm, 4 °C for 10 min, the supernatant was subjected to biochemical analyses. While, 500 µL of sterile saline were used to re-suspend the cell pellets for quantification of the inflammatory cell contents and the total and differential cell counts^61^.

### Assessment of total and differential leucocytic count of BALF pellets

A hemocytometer was used to count the total numbers of cells in the BALF. Smear slides were prepared and stained with Giemsa solution for differential counts of leukocytes in the BALF^61^.

### Biochemical Assessment of BALF supernatant

#### The total protein content

was assessed according to the method of Smith et al.(1985)^62^ using commercial kit (Thermo Scientific, Rockford, USA).

#### The enzymatic LDH activity

was measured using commercial colorimetric kit (Human diagnostics, Wiesbaden, Germany) according to the method of Henry (1974)^63^.

#### Pro-inflammatory cytokine TNF-α and IL-6

levels were analyzed in BALF supernatant using commercially available ELISA kits (R&D Systems, MN, USA). The results are expressed in pg/ml.

#### Brain natriuretic peptide (BNP)

level was assessed in the BALF supernatant using abcam ELISA kit (Cat # ab108816). An ELISA reader was used to measure the optical density at 450 nm, and the results are expressed as ng/ml.

### Histopathological examination

#### Light microscopic examination

Several changes of 70% ethanol were used for washing tissues after lung fixation of the lungs. Afterwards, tissues were subjected to dehydration in ascending alcohol grades, xylene clearing and embedding in paraffin wax to obtain paraffin blocks^64^. Sections of 5 μm thickness were cut, mounted on slides and stained with Hematoxylin and Eosin (H&E) for routine histological examination to study the general histoarchitecture of the lungs^65^, Mallory Trichrome stain for staining the collagen fibers^65^ and Periodic Acid-Schiff (PAS) reaction to quantify the number of mucus-containing goblet cells and mucus expression along bronchial epithelial lining^66^.

#### Immunohistochemical study

For detection of alveolar macrophages (CD68)^67^ and inducible nitric oxide synthase (iNOS)^68^ expression via immunohistochemistry (using the avidin–biotin–peroxidase method), all specimens were processed routinely. Briefly, paraffin sections of 4-μm thickness were dewaxed in xylene, rehydrated in descending series of ethanol, and immersed in 0.3% H_2_O_2_ for 30 min to block endogenous peroxidase. After microwaving of samples for 15 min in citrate buffer (pH 6.0), antigens were retrieved. 10% goat serum (Dako Ltd, Cambridgeshire, UK) was used for 30 min to block non-specific binding. Tissue sections were rinsed gently with PBS and sections were incubated overnight at 4 °C with iNOS antibody (rat monoclonal antibody, 1:500 dilution ,Transduction Laboratories, San Diego, California, USA) and CD68 antibody (mouse monoclonal antibody, 1:200 dilution, code NCL-L-CD68; Leica Biosystems, Benton La, Newcastle Ltd, UK). The sections were incubated with biotinylated anti-rabbit or anti-mouse immunoglobulins (Dako Ltd) for the corresponding primary antibody and thereafter Sections were incubated with the avidin–biotin–horseradish peroxidase complex according to the manufacturer’s instructions (ABC kit from Vector Laboratories Ltd. UK). Peroxides were visualized by incubating the sections in diaminobenzidine (Sigma Chemical Co., Poole, UK) and H_2_O_2_ and counterstained with Mayer’s hematoxylin, dehydrated and mounted with DPX. Negative controls were obtained by neglecting the incubation with the primary antibody. Positive immunoreactivity for CD68 and iNOS staining was defined microscopically by visual identification of brown staining of the immunoreactive cells^67,68^.

#### Histomorphometrical analysis

For the assessment of lung tissue, the following quantitative morphometric parameters were measured using Leica Qwin 500C” software image analyzer computer system (Cambridge, England), in control and experimental groups: the thickness of interalveolar septa in 10 non-overlapping high power fields of H&E stained sections (n = 10) at 400× magnification in all groups^69^. In addition, the mean area percentage of the collagen fibers in sections stained with a Mallory’s trichrome stain was measured^69^ and the mean percentage of PAS-positive goblet cells per bronchiole (number of PAS-positive goblet cells divided by the total epithelial cell number along the basement membrane of randomly selected transversely cut medium sized bronchiole having approximately 100–150 luminal airway epithelial cells) was determined on PAS stained slides for each group^70^. These measurements were taken in 10 non-overlapping fields at 200 × magnification.

Finally, the mean count of alveolar macrophages in anti-CD68 immunostained sections^67^ and the mean area percentage of positive iNOS immunoreactivity in anti-iNOS immunostained sections in 10 non-overlapping high power fields in all groups (n = 10) were assessed too^68^. All these morphometrically measured data were statistically analyzed.

### Statistical analysis

All data were expressed as means ± SD and compared using one-way analysis for variance ANOVA performed with SPSS.22 program (IBM Inc., Chicago, Illinois, USA) followed by Tukey’s post hoc test. The criterion of significance, was set at level of probability (*P* value) less than 0.05.

### Ethics statement

The study was executed in compliance with the ethical guidelines and policies approved by the Animal Care and Use Committee of Faculty of Medicine, AI-Azhar University (Cairo, Egypt) and complies with the Guide for the Care and Use of Laboratory Animals^52^. All measures were taken to decrease the number of animals used and lessen animal suffering.

## Supplementary information

Supplementary figure.

## Data Availability

Supplementary data are available upon request.

## References

[1] Goldberg MA, Antin JH, Guinan EC, Rappeport JM (1986). Cyclophosphamide cardiotoxicity: an analysis of dosing as a risk factor. Blood.

[2] Rehman MU (2012). Cyclophosphamide-induced nephrotoxicity, genotoxicity, and damage in kidney genomic DNA of Swiss albino mice: the protective effect of Ellagic acid. Mol. Cell. Biochem..

[3] Sun Y (2014). Acrolein induced both pulmonary inflammation and the death of lung epithelial cells. Toxicol. Lett..

[4] Mythili Y, Sudharsan PT, Selvakumar E, Varalakshmi P (2004). Protective effect of DL-alpha-lipoic acid on cyclophosphamide induced oxidative cardiac injury. Chem. Biol. Interact..

[5] Boddy AV, Yule SM (2000). Metabolism and pharmacokinetics of oxazaphosphorines. Clin. Pharmacokinet..

[6] Kern JC, Kehrer JP (2002). Acrolein-induced cell death: a caspase-influenced decision between apoptosis and oncosis/necrosis. Chem. Biol. Interact..

[7] Liu Q, Gao Y, Ci X (2019). Role of Nrf2 and its activators in respiratory diseases. Oxid. Med. Cell Longev..

[8] Kumar A, Takada Y, Boriek AM, Aggarwal BB (2004). Nuclear factor-κB: its role in health and disease. J. Mol. Med..

[9] Nagesh R, Kiran Kumar KM, Naveen Kumar M, Patil RH, Sharma SC (2019). Stress activated p38 MAPK regulates cell cycle via AP-1 factors in areca extract exposed human lung epithelial cells. Cytotechnology.

[10] Menendez JT (2016). The mechanism of action of LCZ696. Cardiac. Fail. Rev..

[11] van Veldhuisen DJ (2013). B-type natriuretic peptide and prognosis in heart failure patients with preserved and reduced ejection fraction. J. Am. Coll. Cardiol..

[12] Song Z (2015). Recombinant human brain natriuretic peptide attenuates trauma-/haemorrhagic shock-induced acute lung injury through inhibiting oxidative stress and the NF-kappaB-dependent inflammatory/MMP-9 pathway. Int. J. Exp. Pathol..

[13] Li X, Peng H, Wu J, Xu Y (2018). Brain natriuretic peptide-regulated expression of inflammatory cytokines in lipopolysaccharide (LPS)-activated macrophages via NF-κB and mitogen activated protein kinase (MAPK) pathways. Med. Sci. Monit. Int. Med. J. Exp. Clin. Res..

[14] Wang N (2016). TNF-α-induced NF-κB activation upregulates microRNA-150-3p and inhibits osteogenesis of mesenchymal stem cells by targeting β-catenin. Open Biol..

[15] Zhang D, Lee H, Haspel JA, Jin Y (2017). Long noncoding RNA FOXD3-AS1 regulates oxidative stress-induced apoptosis via sponging microRNA-150. FASEB J. Off. Publ. Fed. Am. Soc. Exp. Biol..

[16] Tripathi DN, Jena GB (2008). Astaxanthin inhibits cytotoxic and genotoxic effects of cyclophosphamide in mice germ cells. Toxicology.

[17] El-Sheikh AA, Morsy MA, Okasha AM (2017). Inhibition of NF-kappaB/TNF-alpha pathway may be involved in the protective effect of resveratrol against cyclophosphamide-induced multi-organ toxicity. Immunopharmacol. Immunotoxicol..

[18] Judge P, Haynes R, Landray MJ, Baigent C (2015). Neprilysin inhibition in chronic kidney disease. Nephrol. Dial. Transpl. Off. Publ. Eur. Dial. Transpl. Assoc. Eur. Renal Assoc..

[19] Yang X, Zhang Q, Gao Z, Yu C, Zhang L (2018). Down-regulation of MiR-150 alleviates inflammatory injury induced by interleukin 1 via targeting Kruppel-like factor 2 in human chondrogenic cells. Cell. Physiol. Biochem. Int. J. Exp. Cell. Physiol. Biochem. Pharmacol..

[20] Yang H (2014). Protective effect of rhBNP on intestinal injury in the canine models of sepsis. Int. Immunopharmacol..

[21] Rubattu S, Forte M, Marchitti S, Volpe M (2019). Molecular implications of natriuretic peptides in the protection from hypertension and target organ damage development. Int. J. Mol. Sci..

[22] Behrend L, Henderson G, Zwacka RM (2003). Reactive oxygen species in oncogenic transformation. Biochem. Soc. Trans..

[23] Macfarlane LA, Murphy PR (2010). MicroRNA: biogenesis, function and role in cancer. Curr. Genom..

[24] Wang J (2018). MicroRNA-143 modulates the expression of natriuretic peptide receptor 3 in cardiac cells. Sci. Rep..

[25] Ismail M, Hossain MF, Tanu AR, Shekhar HU (2015). Effect of spirulina intervention on oxidative stress, antioxidant status, and lipid profile in chronic obstructive pulmonary disease patients. Biomed. Res. Int..

[26] El-Agamy DS, Elkablawy MA, Abo-Haded HM (2017). Modulation of cyclophosphamide-induced cardiotoxicity by methyl palmitate. Cancer Chemother. Pharmacol..

[27] Chakraborty P (2009). Modulation of cyclophosphamide-induced cellular toxicity by diphenylmethyl selenocyanate in vivo, an enzymatic study. J. Cancer Mol..

[28] Nagaraj S (2012). Antiproliferative potential of astaxanthin-rich alga Haematococcus pluvialis Flotow on human hepatic cancer (HepG2) cell line. Biomed. Prev. Nutr..

[29] Jing W (2017). LCZ696 (Sacubitril/valsartan) ameliorates oxidative stress, inflammation, fibrosis and improves renal function beyond angiotensin receptor blockade in CKD. Am. J. Transl. Res..

[30] Pramanik KC, Makena MR, Bhowmick K, Pandey MK (2018). Advancement of NF-κB signaling pathway: a novel target in pancreatic cancer. Int. J. Mol. Sci..

[31] Giebelen IA, van Westerloo DJ, LaRosa GJ, de Vos AF, van der Poll T (2007). Local stimulation of alpha7 cholinergic receptors inhibits LPS-induced TNF-alpha release in the mouse lung. Shock (Augusta, Ga.).

[32] Abdelaziz RR, Elkashef WF, Said E (2015). Tranilast reduces serum IL-6 and IL-13 and protects against thioacetamide-induced acute liver injury and hepatic encephalopathy. Environ. Toxicol. Pharmacol..

[33] Paulsen CE (2011). Peroxide-dependent sulfenylation of the EGFR catalytic site enhances kinase activity. Nat. Chem. Biol..

[34] Hao Y (2014). Mycoplasma pneumoniae modulates STAT3- STAT6/EGFR-FOXA2 signaling to induce overexpression of airway mucins. Infect. Immun..

[35] Harris JF (2005). Bcl-2 sustains increased mucous and epithelial cell numbers in metaplastic airway epithelium. Am. J. Respir. Crit. Care Med..

[36] Takeyama K (2008). Role of epidermal growth factor receptor in maintaining airway goblet cell hyperplasia in rats sensitized to allergen. Clin. Exp. Allergy.

[37] Wang Z (2014). Corilagin attenuates aerosol bleomycin-induced experimental lung injury. Int. J. Mol. Sci..

[38] He YM, Zhu S, Ge YW, Cai SQ, Komatsu K (2015). Secoiridoid glycosides from the root of *Gentiana**crassicaulis* with inhibitory effects against LPS-induced NO and IL-6 production in RAW264 macrophages. J. Nat. Med..

[39] Ahmed SM, Luo L, Namani A, Wang XJ, Tang X (2017). Nrf2 signaling pathway: pivotal roles in inflammation. Biochim. Biophys. Acta Mol. Basis Dis..

[40] Hwang Y-J (2013). MafK positively regulates NF-κB activity by enhancing CBP-mediated p65 acetylation. Sci. Rep..

[41] Ullah MF (2015). Sulforaphane (SFN): an isothiocyanate in a cancer chemoprevention paradigm. Medicines (Basel, Switzerland).

[42] Li W (2008). Activation of Nrf2-antioxidant signaling attenuates NFkappaB-inflammatory response and elicits apoptosis. Biochem. Pharmacol..

[43] Kumar S, Boehm J, Lee JC (2003). p38 MAP kinases: key signalling molecules as therapeutic targets for inflammatory diseases. Nat. Rev. Drug Discov..

[44] Tanel A, Averill-Bates DA (2007). P38 and ERK mitogen-activated protein kinases mediate acrolein-induced apoptosis in Chinese hamster ovary cells. Cell. Signal..

[45] Iborra-Egea O (2017). Mechanisms of action of sacubitril/valsartan on cardiac remodeling: a systems biology approach. NPJ Syst. Biol. Appl..

[46] Mezzasoma L, Antognelli C, Talesa VN (2017). A novel role for brain natriuretic peptide: inhibition of IL-1beta secretion via downregulation of NF-kB/Erk 1/2 and NALP3/ASC/Caspase-1 activation in human THP-1 monocyte. Mediat. Inflamm..

[47] Wan Y (2017). Identification of four oxidative stress-responsive microRNAs, miR-34a-5p, miR-1915-3p, miR-638, and miR-150-3p, in hepatocellular carcinoma. Oxid. Med. Cell. Longev..

[48] Qin B (2017). MicroRNA-150 targets ELK1 and modulates the apoptosis induced by ox-LDL in endothelial cells. Mol. Cell. Biochem..

[49] Xue H, Li MX (2017). MicroRNA-150 protects against cigarette smoke-induced lung inflammation and airway epithelial cell apoptosis through repressing p53: microRNA-150 in CS-induced lung inflammation. Hum. Exp. Toxicol..

[50] Wang N (2016). TNF-alpha-induced NF-kappaB activation upregulates microRNA-150-3p and inhibits osteogenesis of mesenchymal stem cells by targeting beta-catenin. Open Biol..

[51] Cao M (2014). miR-150 promotes the proliferation and migration of lung cancer cells by targeting SRC kinase signalling inhibitor 1. Eur. J. Cancer (Oxford, England: 1990).

[52] National Research Council (US) Institute for Laboratory Animal Research. *Guide**for**the**Care**and**Use**of**Laboratory**Animals*. National Academies Press (US), Washington (DC) (1996). 25121211

[53] Ahmed LA, El-Maraghy SA, Rizk SM (2015). Role of the KATP channel in the protective effect of nicorandil on cyclophosphamide-induced lung and testicular toxicity in rats. Sci. Rep..

[54] Razak RNHA (2019). Ameliorative effects of *Aquilaria**malaccensis* leaves aqueous extract on reproductive toxicity induced by cyclophosphamide in male rats. Malays. J. Med. Sci..

[55] Imran M (2019). Sacubitril and valsartan protect from experimental myocardial infarction by ameliorating oxidative damage in Wistar rats. Clin. Exp. Hypertens..

[56] Nassef NA (2019). Quercetin improves platelet function and ultrastructure in cholestatic liver injury in rats: Role of ORAI1 gene expression. Gene Rep..

[57] Jin M, Yang Z, Ye W, Xu H, Hua X (2014). MicroRNA-150 predicts a favorable prognosis in patients with epithelial ovarian cancer, and inhibits cell invasion and metastasis by suppressing transcriptional repressor ZEB1. PLoS ONE.

[58] Eshra MA, Rashed LA, Eltelbany RF, Omar H, Shams Eldeen AM (2019). Omega-3 modulates anxiety and improves autistic like features induced by high fat diet but not valproate. Neurol. Psychiatry Br. Res..

[59] Mohammed MA, Aboulhoda BE, Mahmoud RH (2018). Vitamin D attenuates gentamicin-induced acute renal damage via prevention of oxidative stress and DNA damage. Hum. Exp. Toxicol..

[60] Pederson P, Bollag DM, Rozycki MD, Edelstein SJ (1997). Protein methods. Proteins: Structure, Function, and Bioinformatics.

[61] Verma R, Brahmankar M, Kushwah L, Suresh B (2013). Evaluating the inhibitory potential of sulindac against the bleomycin-induced pulmonary fibrosis in wistar rats. Environ. Toxicol. Pharmacol..

[62] Smith PK (1985). Measurement of protein using bicinchoninic acid. Anal. Biochem..

[63] Henry R (1974). Colorimetric Determination of Lactic Dehydrogenase.

[64] Carson FL, Cappellano CH (2009). Histotechnology: A Self Instructional Text.

[65] Bancroft M, Gamble M (2008). Theory and Practice of Histological Techniques.

[66] Suvarna K, Layton C, Bancroft J (2013). Theory and Practice of Histological Techniques.

[67] Ramos Vara JA (2008). American association of veterinary laboratory diagnosticians subcommittee on standardization of immunohistochemistry: suggested guidelines for immunohistochemical techniques in veterinary diagnostic laboratories. J. Vet. Diagn. Invest..

[68] Vinas JL (2006). NO and NOS isoforms in the development of apoptosis in renal ischemia/reperfusion. Free Radic. Biol. Med..

[69] Izbicki G, Segel MJ, Christensen TG (2002). Time course of bleomycin-induced lung fibrosis. Int. J. Exp. Pathol..

[70] Cho JY, Miller M, Baek KJ (2001). Immunostimulatory DNA sequences inhibit respiratory syncytial viral load, airway inflammation, and mucus secretion. J. Allergy Clin. Immunol..

