# Supplementary material for: A novel protective role of sacubitril/valsartan in cyclophosphamide induced lung injury in rats: impact of miRNA-150-3p on NF-κB/MAPK signaling trajectories
Source: Sci Rep. 2020 Aug 3;10:13045. doi: 10.1038/s41598-020-69810-5 (PMC7400763; doi:10.1038/s41598-020-69810-5)
Supplement: Supplementary file 1 — Supplementary figure. [file 41598_2020_69810_MOESM1_ESM.pdf]

A novel protective role of sacubitril/valsartan in cyclophosphamide induced lung injury in rats: Impact of miRNA-150-3p on NF-κB/MAPK signaling trajectories

Ghada A. Abdel-Latif [a,h]*, Azza H. Abd Elwahab [b], Rehab A. Hasan [c], Noura F. ElMongy [d], Maggie M. Ramzy [e], Manal L. Louka [f], Mona F. Schaalan[g,h]

[a] *Pharmacology & Toxicology Department, Faculty of Pharmacy, Misr International University (MIU), Cairo, Egypt*
[b] *Physiology Department, Faculty of Medicine for Girls, Al-Azhar University, Cairo, Egypt*
[c] *Histology Department, Faculty of Medicine for Girls, Al-Azhar University, Cairo, Egypt*
[d] *Physiology Department, Faculty of Medicine, Al-Azhar University, Damietta, Egypt*
[e] *Biochemistry Department, Faculty of Medicine, Minia University, Egypt*
[f] *Medical Biochemistry department, Faculty of medicine, Ain Shams University, Cairo, Egypt*
[g] *Pharmacy Practice & Clinical Pharmacy Department, Faculty of Pharmacy, Misr International University (MIU), Cairo, Egypt*
[h] *Translational and Clinical Research Unit, Faculty of Pharmacy, Misr International University (MIU), Cairo, Egypt*

P-P38

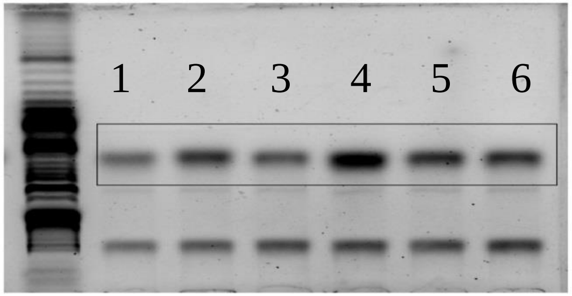

T-P38

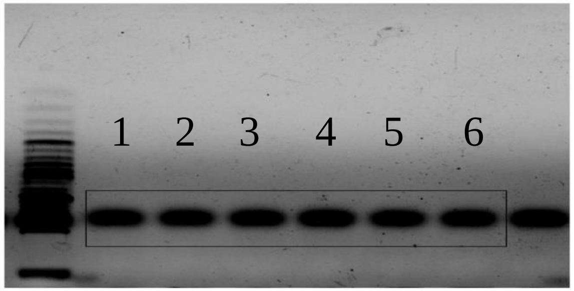

P-ERK1/2

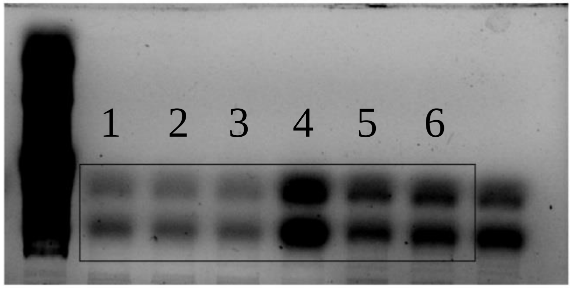

T-ERK1/2

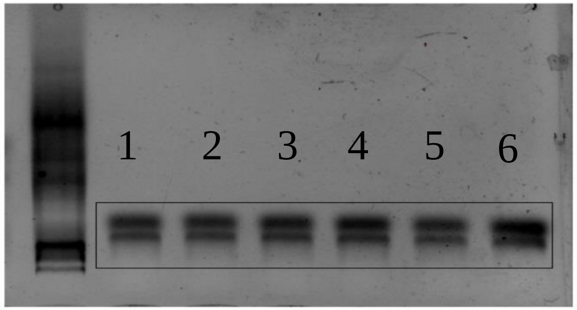

B-actin

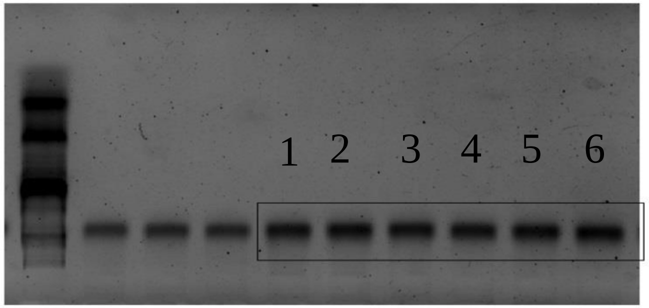

<u>Key for blots images:</u>

1- Control
2- ENT
3- VAL
4- CP
5- ENT + CP
6- VAL + CP
